# Zingerone Mitigates Testicular Dysfunction Induced by Cisplatin

**DOI:** 10.5935/1518-0557.20250181

**Published:** 2026

**Authors:** Elham Younesi, Layasadat Khorsandi, Amirhesam Keshavarz Zarjani, Abbas Heidari-Moghadam, Mohammad Javad Khodayar, Yousef Asadi-Fard

**Affiliations:** 1 Student Research committee, Ahvaz Jundishapur University of Medical Sciences, Ahvaz, Iran; 2 Cellular and Molecular Research Center, Medical Basic Sciences Research Institute, Ahvaz Jundishapur University of Medical Sciences, Ahvaz, Iran; 3 Department of Anatomical Sciences, Faculty of Medicine, Ahvaz Jundishapur University of Medical Sciences, Ahvaz, Iran; 4 Department of Anatomical Sciences, School of Medicine, Dezful University of Medical Sciences, Dezful, Iran; 5 Toxicology Research Center, Medical Basic Sciences Research Institute, Ahvaz Jundishapur University of Medical Sciences, Ahvaz, Iran; 6 Department of Anatomy, School of Medicine, Arak University of Medical Sciences, Arak, Iran

**Keywords:** zingerone, cisplatin, oxidative stress, apoptosis, reproductive system

## Abstract

**Objective:**

Cisplatin is one of the most widely used antitumor drugs globally, particularly in treating various solid tumors. The reproductive system is impacted by cisplatin toxic effects. This study aims to understand how Zingerone affects spermatogenesis defects in mice.

**Methods:**

In the present experimental laboratory study, the 48 male NMRI mice (6 to 8 weeks of age, 25 to 30g weight) were treated with Cisplatin (7 mg/kg) for 5 days and zingerone for 30 days at concentrations of 10, 20, and 40 mg/kg before cisplatin administration. After the treatment period, the testicles were dissected immediately following sacrifice. Morphometric parameters, serum testosterone concentration, histology, Bax/Bcl-2 ratio, and testis weight have been assessed. To determine levels of oxidative stress, malondialdehyde contents and antioxidant levels were evaluated.

**Results:**

Cisplatin-induced structural damages enhanced the Bax/Bcl-2 ratio, and reduced testosterone levels and testis weight. Cisplatin caused oxidative stress by enhancing malondialdehyde contents in the mouse testicles. Zingerone dose-dependently reduced the Bax/Bcl-2 ratio and reversed the histological changes, testosterone levels, and antioxidant capacity.

**Conclusions:**

According to the results of the present study, Pretreatment with zingerone can improve testosterone production by preventing apoptosis and oxidative stress in the testicles of mice that have undergone cisplatin intoxication.

## INTRODUCTION

Infertility is one of the main critical issues associated with cancer treatment (Tian En *et al*., 2020). In young male patients, chemotherapy agents affect fertility by impairing testicular function (Okada & Fujisawa, 2019; Yumura *et al*., 2023). Cisplatin (CP) is used to manage various malignancies, such as testicular and ovarian cancers (Helm & States, 2009; Zoń & Bednarek, 2023). CP induces apoptosis by generating oxidative stress and interacting with DNA (Dermitzakis *et al*., 2016). It causes testicular damage characterized by germ cell apoptosis, impaired steroidogenesis, and histological alterations (A A Aly & Eid, 2020; Nna *et al*., 2020; Saher *et al*., 2023). Infertility can result from CP treatment due to its damaging effects on Leydig cells, which are crucial for testosterone production. This impairment in testosterone synthesis disrupts spermatogenesis (Datrianto *et al*., 2021; Demir & Altındağ, 2022). Oxidative stress is a key factor that regulates apoptosis, and various mechanisms of apoptosis modulation by oxidative stress have been established (Sharma *et al*., 2023).

According to studies, apoptosis plays a crucial role in regulating testicular function (Rotimi & Singh, 2022). Excessive apoptosis in spermatogenic cells can lead to infertility (Vardiyan *et al*., 2020). The primary regulators of cell survival and apoptosis are Bcl-2 and Bax (Hatok & Racay, 2016). Extracting bioactive molecules from plants can improve sperm quality, testosterone secretion, and fertility indices (Shahedi *et al*., 2021; Yaghutian Nezhad *et al*., 2021; Behairy *et al*., 2022; Rotimi *et al*., 2023). Among many natural substances, flavonoids have garnered significant attention for their potential to treat male reproductive system dysfunction (Farombi *et al*., 2012; Ye *et al*., 2020).

Flavonoids, as secondary metabolites of polyphenolic plants and fungi, possess a phenyl-benzopyran structure. They are natural antioxidants with potential benefits such as anti-inflammatory, immune-stimulating, antiviral, anticancer, anti-apoptotic, and anti-allergic properties (Al-Khayri *et al*., 2022). Ginger, one of the most commonly used herbs, is a traditional medicine employed globally (El-Seedi *et al*., 2019). Ginger contains a significant amount of Zingerone (ZG), which is believed to be responsible for its pharmacological properties. ZG exhibits potent antioxidant, anti-inflammatory, anticancer, antidiabetic, antihypertensive, antimicrobial, antithrombotic, anxiolytic, anti-ulcer, and appetite-stimulant properties (Ahmad *et al*., 2015).

In addition to its antioxidant properties, ZG is used to treat various diseases due to its ability to scavenge free radicals. As a result, ZG can reduce reactive oxygen species (ROS) while maintaining its antioxidant qualities. ZG mitigates cisplatin-induced kidney damage by attenuating oxidative stress, suppressing apoptotic gene expression, and reducing inflammatory factors (Kandemir *et al*., 2019). In a rat model of CIS-induced nephrotoxicity, ZG demonstrated nephroprotective effects, primarily by suppressing oxidative stress and inflammation (Alibakhshi *et al*., 2018). It is essential to identify a compound that can mitigate the adverse effects of CP on the human reproductive system. To date, the effects of Zingerone on cisplatin-induced cytotoxicity have not been thoroughly investigated. The purpose of this research is to analyze how ZG protects against CP-induced testicular toxicity in mice by evaluating oxidative stress and apoptosis.

## MATERIAL AND METHODS

### Animals and Research Design (Figure 1)

For this experiment, a total of 48 adult male NMRI mice, aged 6 to 8 weeks and weighing between 25 to 30 grams each, were purchased from the Animal Care Center in Ahvaz, Iran. The animals were housed under standard laboratory conditions with a humidity level of 50±5%, a 12-hour light/dark cycle, and a temperature of 22±5 °C throughout the study. The research was conducted in 2023 at the Cellular and Molecular Research Center of Ahvaz Jundishapur University of Medical Sciences, Ahvaz, Iran. This project adhered to the guidelines established by the Animal Ethics Committee of our institution (approval code: IR.AJUMS.ABHC.REC.1402.006).

**Figure 1:**
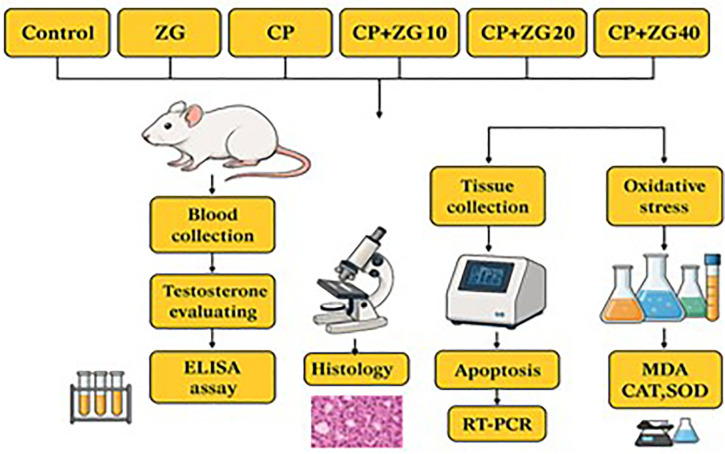
Schematic illustration detailing the experimental design of the study.

The mice were randomly assigned to six groups (eight animals per group), as described below:

- Control: Received 0.2 ml of normal saline intraperitoneally (IP) for 35 days.
- CP: Administered 7 mg/kg of cisplatin (CP) intraperitoneally (IP) on days 30-35.
- ZG: received Zingerone (ZG) orally at a dose of 40 mg/kg for 35 days.
- CP+ZG (10, 20 and 40): Received ZG orally at doses of 10, 20, or 40 mg/kg for 35 days, along with 7 mg/kg CP (IP) on days 30-35.

ZG and CP were purchased from Sigma Company. Both compounds were dissolved in normal saline. The dosing protocols for ZG (Sigma) and CP (Sigma) were based on previous studies (Aldemir *et al*., 2014; Hassan *et al*., 2019). One day after the final treatment, the mice were euthanized using high doses of ketamine and xylazine. Following euthanasia, the testes were promptly dissected. The left testes were fixed in Bouin’s solution for histological examination and right testes were stored at -80°C for other assessments, while the left testes were fixed in Bouin’s solution for histological examination.

### Histology and Morphometry

Six microscopy slides per animal were stained with hematoxylin and eosin (H&E) to analyze histological criteria, such as detachment of germ cells and vacuolization of the germinal epithelium. The average percentage of each characteristic was calculated for every treatment group. Two researchers, blinded to the group allocation, independently analyzed the slides. The diameter of the seminiferous tubules and the height of the seminiferous epithelium were calculated using Motic software. For each animal in the experiment, the researchers assessed 100 tubules (Bahrami *et al*., 2018).

### Evaluation of lipid peroxidation

After centrifuging the testicular homogenate, 500 µL of the supernatant was mixed with 10% trichloroacetic acid and centrifuged again at 7000×g for 10 minutes. The resulting supernatant was then transferred to two milliliters of trichloroacetic acid. Each sample was centrifuged at 7500×g for 10 minutes after adding N-butanol. The optical density (OD) of the supernatant was measured at 535 nm using a spectrophotometer.

### SOD and CAT activity

The Ransod kit (SD125, United Kingdom) was used to determine superoxide dismutase (SOD) activity. This method involves the reaction with the superoxide anion, producing a water-soluble formazan dye. SOD inhibits xanthine oxidase, creating a linear relationship between its activity and the rate of reduction. The inhibition activity of SOD was measured at 505 nm using a spectrophotometer. Catalase (CAT) activity was assessed by measuring the rate of complex formation between hydrogen peroxide and ammonium molybdate.

### Testosterone evaluating

Blood samples were collected directly from the heart using heparinized tubes, then centrifuged at 400 g for 20 minutes to separate the serum. After centrifugation, the samples were stored at -80°C until analysis. A mouse testosterone ELISA assay kit was utilized to detect serum testosterone levels (Monobind, USA).

### Real-time PCR

RNA was extracted from testicular tissue using the RNeasy kit (Qiagen, Germany). To synthesize complementary DNA (cDNA), the extracted RNA was reverse-transcribed using a reverse transcription kit (Qiagen, Germany). The PCR reaction mixture consisted of SYBR Green Master Mix (Qiagen), DEPC-treated water, cDNA, and forward and reverse primers. Real-time PCR (RT-PCR) was performed over 45 cycles, with the following steps: initial denaturation at 95°C for 50 seconds, followed by 45 cycles of denaturation at 95°C for 30 seconds, and annealing/extension at 60°C for 35 seconds. The relative gene expression levels were normalized to GAPDH as the reference gene. Data analysis was conducted using REST software (2009).

### Statistical analyses

Statistical analyses were conducted using the Statistical Package for the Social Sciences (SPSS) software (version 21.0, Chicago, IL, USA). Data were evaluated through one-way analysis of variance (ANOVA), followed by post hoc Tukey’s or LSD tests. Statistical significance was determined at a *p*-value<0.05, and results were expressed as mean±standard deviation (SD).

## RESULTS

### Body and relative testis weight

**Figure 2:**
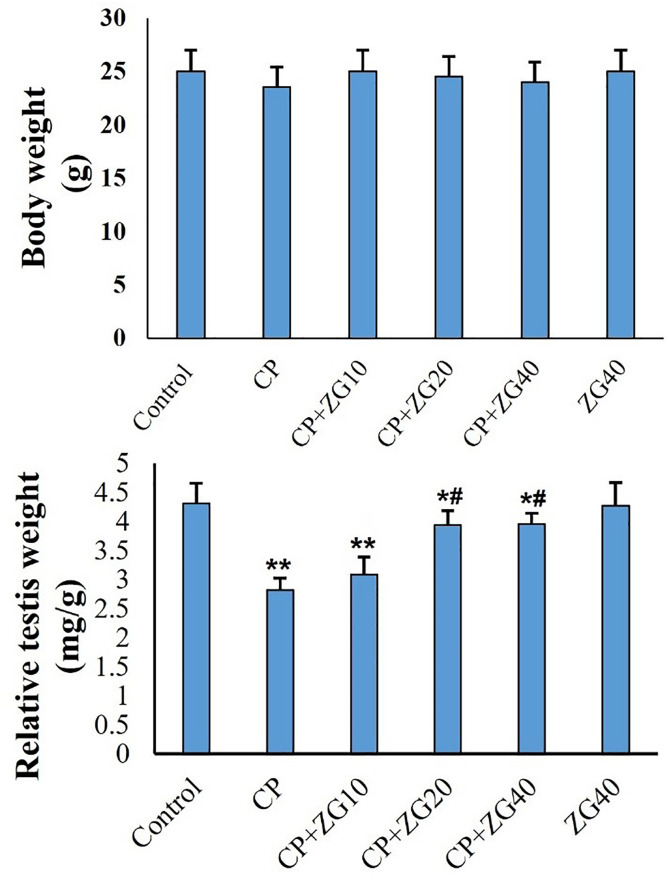
Effects of ZG on (A) body weight and (B) relative testes weight in CP-treated mice. Data are illustrated in mean±SD (n=6). *p<0.05, **p<0.01, ^#^*p*<0.05. Symbols indicate comparison to the control (*) and CP (#) groups (one-way ANOVA followed by Tukey’s post-hoc test).

Body weights did not significantly differ among the groups. However, mice exposed to cisplatin (CP) showed a significant decrease in the ratio of testis weight to body weight (relative testis weight) compared to the control group (*p*<0.01). Treatment with Zingerone (ZG) dose-dependently increased the relative testis weight in CP-treated animals (*p*<0.05, Figure 2).

### Testosterone assay

**Figure 3:**
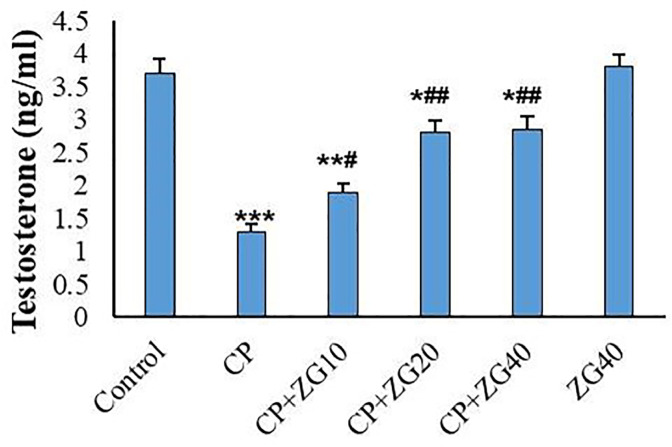
Effects of ZG on serum testosterone concentration in CP-treated mice. CP administration significantly decreased testosterone levels (***p<0.001 vs control). Co-treatment with ZG at 20 and 40 mg/kg (CP +ZG20 and CP +ZG40 groups) significantly restored testosterone levels compared to CP group (##p<0.01), while CP +ZG10 showed a modest increase. Data are presented as mean±SD (n=6). *p<0.05, **p<0.01, ***p<0.001 vs control group; ^#^*p*<0.05, ^##^*p*<0.01 *vs* CP group (one-way ANOVA followed by Tukey’s post-hoc test).

In CP-treated mice, testosterone levels were significantly reduced (*p*<0.001). However, testosterone levels were significantly increased in the CP+ZG20 and CP+ZG 40 groups compared to the CP-only group (*p*<0.01). The CP+ZG10 group also showed a slight but significant increase in testosterone levels compared to the CP-treated mice (*p*<0.05, Figure 3).

### Histology and Morphometry

**Figure 4:**
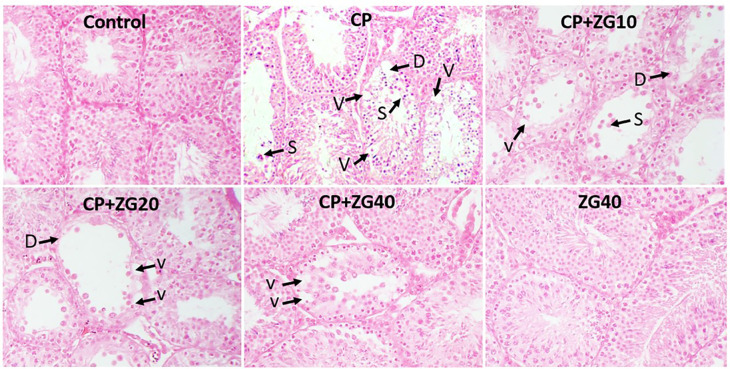
Photomicrograph from testicular tissues. Control group exhibits typical seminiferous epithelium; CP group shows vacuolization (V) and sluing (S) of the germinal epithelium, and extensive degenerative changes (D); The CP+ZG10 and CP+ZG20 groups shows moderate degenerative changes and vacuolization in g; and CP+ZG40 group displays minimal degenerative changes and vacuolization in the germinal epithelium. ZG40 group shows typical germinal epithelium. H&E staining; Magnifications: ×250.

**Figure 5:**
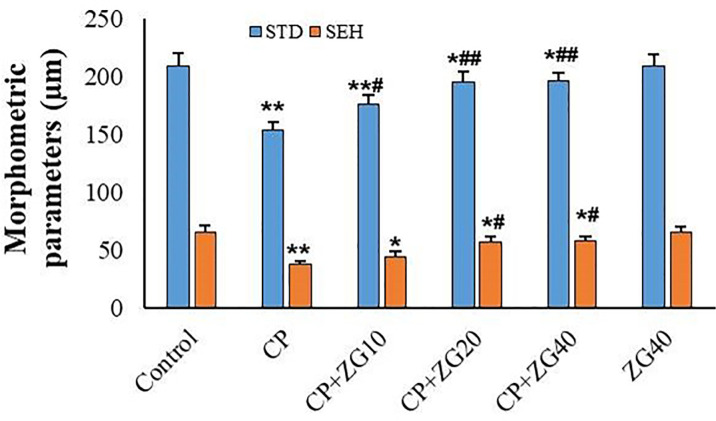
Histopathological evaluation of testicular tissue in: Control, CP-treated, CP +ZG10, CP +ZG 20, CP +ZG40, and ZG-alone groups. Two blinded investigators independently analyzed 100 seminiferous tubules per animal using Motic software to measure tubule diameter and epithelial height. Data are presented as mean±SD (n=6 groups). *p<0.05 and **p<0.01 vs control group; ^#^*p*<0.05 and ^##^*p*<0.01 *vs* CP group (one-way ANOVA with Tukey’s post-hoc test).

The control and ZG40 groups displayed normal histological features, characterized by well-preserved seminiferous epithelium. In contrast, CP-treated mice exhibited sloughing and vacuolization of the seminiferous tubules, leading to atrophy of the germinal epithelium. Treatment with ZG restored normal tissue architecture and seminiferous parameters, as illustrated in Figures 4 and 5.

### Expression of apoptosis-related genes

**Figure 6:**
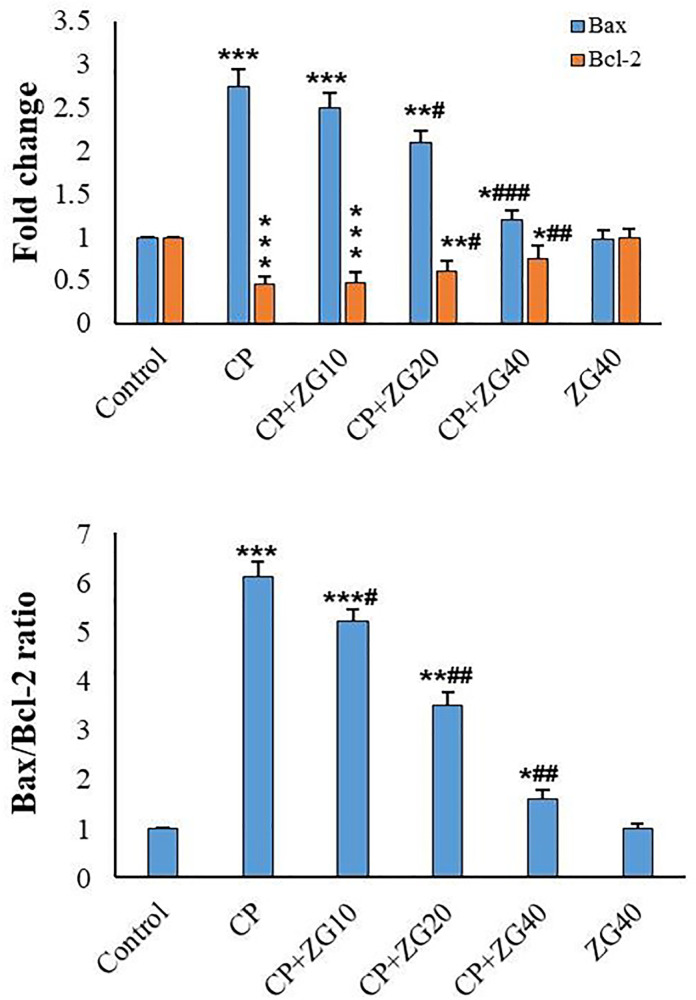
Effects of ZG on the Bax/Bcl-2 ratio in testicular tissue of CP-treated mice. The Bax/Bcl-2 ratio was significantly elevated in CP-treated mice compared to controls (***p<0.001). ZG co-treatment at 20 and 40 mg/kg (CP +ZG20 and CP +ZG40 groups) significantly reduced the ratio compared to CP-treated mice (^##^*p*<0.01), with CP +ZG40 showing the most pronounced effect. Notably, the ratio in CP +ZG20 and CP +ZG40 groups was significantly lower than in CP +ZG10 group (*p*<0.05). Data represent mean±SD (n=6 groups). *p<0.05, **p<0.01, ***p<0.001 *vs*. control; ^#^*p*<0.05, ^##^*p*<0.01, ^###^*p*<0.001 *vs*. CP group (one-way ANOVA with Tukey’s post-hoc test).

The Bax/Bcl-2 ratio was significantly increased in CP-treated mice (*p*<0.001). However, a significant reduction in the Bax/Bcl-2 ratio was observed in the CP+ZG20 and CP+ZG40 groups compared to the CP-treated group (*p*<0.01). Furthermore, the Bax/Bcl-2 ratio in the CP+ZG20 and CP+ZG40 groups was significantly lower than that in the CP+ZG10 group (Figure 6).

### MDA and antioxidant levels

**Figure 7:**
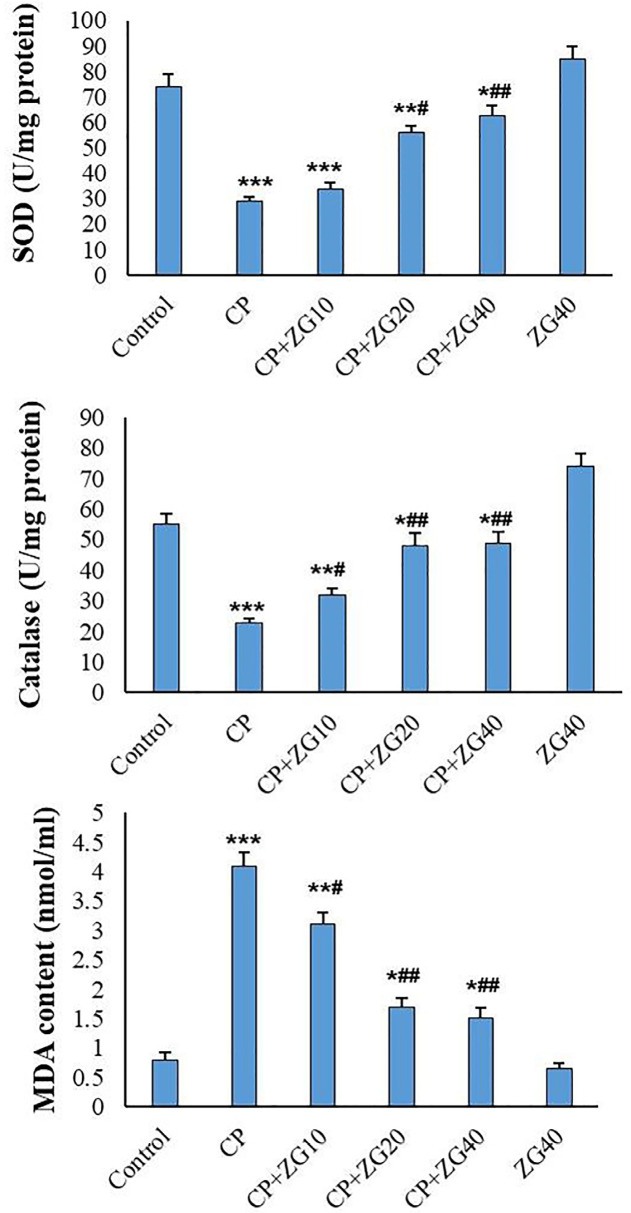
Effects of ZG on oxidative stress markers in testicular tissue of CP-treated mice. SOD activity, CAT activity, and MDA levels. CP administration significantly reduced SOD and CAT activities (*p*<0.001 *vs*. control) while increasing MDA content (*p*<0.001). ZG pretreatment at 20 and 40 mg/kg (CP +ZG20 and CP +ZG40 groups) significantly restored SOD (^#^*p*<0.05) and CAT activity (^##^*p*<0.01), and dose-dependently reduced MDA levels (^#^*p*<0.05, ^##^*p*<0.01) compared to CP group. ZG alone showed no significant effects on antioxidant indices versus controls. Data represent mean±SD (n=6 groups). *p<0.05, **p<0.01, ***p<0.001 *vs*. control group; ^#^*p*<0.05, ^##^*p*<0.01 *vs*. CP group (one-way ANOVA with Tukey’s post-hoc test).

Administration of CP significantly reduced testicular SOD and CAT enzyme activity compared to the control group (*p*<0.001). Additionally, MDA levels were significantly increased in the CP group compared to the control animals (*p*<0.001). Pre-treatment with ZG20 and ZG40 significantly increased testicular SOD and CAT levels compared to the CP group (*p*<0.05 and *p*<0.01, respectively). However, administration of ZG to normal animals did not result in significant changes in antioxidant indices compared to the control group. In CP-treated mice, ZG administration dose-dependently reduced testicular MDA levels compared to the CP group (Figure 7).

## DISCUSSION

This study aimed to investigate the protective effects of ZG against CP-induced testicular damage. Our findings demonstrated that CP caused structural changes in testicular tissue, increased cell death and oxidative stress, and reduced testosterone levels and testicular weights. However, pre-treatment with ZG significantly mitigated these adverse effects in a dose-dependent manner.

The reduction in testicular weights highlights the toxic effects of CP on mouse testes. The reversal of testis weight following ZG administration suggests that ZG may prevent CP-induced germ cell loss. This hypothesis is supported by morphometric findings. CP treatment reduced the diameter of seminiferous tubules and the height of the germinal epithelium, whereas ZG treatment improved these morphometric parameters in CP-injected mice, indicating enhanced spermatogenesis. These results align with previous studies (Abdullah & Bondagji, 2011; Yahyazadeh *et al*., 2020).

In a previous study, ZG was shown to improve sperm quality, reduce malondialdehyde (MDA) levels, and increase superoxide dismutase (SOD) and glutathione (GSH) levels in testicular tissue (Rafiee *et al*., 2019). In the current study, ZG pretreatment effectively reduced MDA levels in the testicles of CP-treated mice. Similarly, Mohammadi *et al*. (2014) demonstrated that ZG alleviated oxidative damage in testicular tissue induced by cyclophosphamide. Additionally, Çağlayan *et al*. (2019) reported that ZG administration improved the oxidative/antioxidative balance, enhanced sperm quality, and increased testosterone levels in vancomycin-intoxicated subjects.

Recent studies have elucidated that cisplatin (CP)-induced testicular toxicity primarily involves three interlinked molecular pathways: CP administration triggers NADPH oxidase (NOX4)-dependent ROS overproduction, depleting antioxidant defenses (SOD2, CAT) and impairing steroidogenic enzymes (StAR, CYP11A1), ultimately reducing testosterone synthesis, CP upregulates pro-apoptotic Bax while suppressing anti-apoptotic Bcl-2 in germ cells, leading to cytochrome c release and caspase-3 activation and Disruption of Steroidogenesis (Cregan *et al*., 2013; A A Aly & Eid, 2020; Sharma *et al*., 2023).

Lowered levels of testosterone in the CP group could indicate damage to Leydig cells and dysfunction in steroidogenesis. Leydig cells, which are responsible for testosterone production, are particularly vulnerable to oxidative stress (Awny *et al*., 2021). Previous studies have demonstrated that oxidative stress induces apoptosis in Leydig cells (Sun *et al*., 2017). Therefore, the observed decrease in testosterone levels following CP exposure may be attributed to Leydig cell damage.

The seminiferous tubules exhibit histological changes as a result of reduced testosterone levels (Asadi *et al*., 2017). Additionally, it has been established that declining testosterone levels can lead to the induction of apoptosis in germ cells (Yadav *et al*., 2022). The presence of vacuoles in the germinal epithelium may indicate germ cell apoptosis caused by CP (Hasanin *et al*., 2018). This hypothesis is further supported by the increased Bax/Bcl-2 ratio in the testicular tissue. Mesbahzadeh *et al*. (2021) found that CP exposure reduced tubular diameter, testosterone levels, and increased seminiferous tubule apoptosis in rats. However, ZG was able to reverse these morphometric parameters, suggesting a reduction in germ cell apoptosis in CP-treated mice.

Following testicular damage induced by zinc oxide nanoparticles, Rafiee *et al*. (2019) observed that ZG improved histological changes and reduced apoptosis in testicular tissue. In this study, ZG demonstrated the potential to elevate testosterone levels, reduce vacuolarization in the seminiferous tubules, and decrease the Bax/Bcl-2 ratio. These findings suggest that ZG may attenuate CP-induced testicular damage by inhibiting germ cell apoptosis. This study was intentionally designed to evaluate ZG testicular protective effects; thus, systematic evaluation of off-target actions in non-reproductive tissues was not included. Future studies should address ZG organ-specific pharmacokinetics and potential dose-related adverse effects to fully establish its clinical translational potential.

## CONCLUSION

This investigation has demonstrated that ZG attenuates testicular injury induced by CP in mice. ZG pretreatment increases testosterone levels, enhances antioxidant capacity, and suppresses apoptosis. Further research is necessary to elucidate the specific mechanisms by which ZG mitigates CP-induced toxicity.
